# Development of a complex community pharmacy intervention package using theory-based behaviour change techniques to improve older adults’ medication adherence

**DOI:** 10.1186/s12913-020-05282-7

**Published:** 2020-05-13

**Authors:** D. E. Patton, C. Ryan, C. M. Hughes

**Affiliations:** 1 School of Pharmacy, Queen’s University Belfast, Belfast, Northern Ireland; 2 School of Pharmacy and Pharmaceutical Sciences, Trinity College Dublin, Dublin, Ireland

**Keywords:** Theoretical domains framework, Behaviour change techniques, Intervention development, Complex intervention, Tailored, Medication adherence, Community pharmacy

## Abstract

**Background:**

To improve the effectiveness of interventions targeting non-adherence in older adults, a systematic approach to intervention design is required. The content of complex interventions and design decisions are often poorly described in published reports which makes it difficult to explore why they are ineffective. This intervention development study reports on the design of a community pharmacy-based adherence intervention using 11 Behaviour Change Techniques (BCTs) which were identified from previous qualitative research with older patients using the Theoretical Domains Framework.

**Methods:**

Using a group consensus approach, a five-step design process was employed. This focused on decisions regarding: (1) the overall delivery format, (2) formats for delivering each BCT; (3) methods for tailoring BCTs to individual patients; (4) intervention structure; and (5) materials to support intervention delivery. The APEASE (Affordability; Practicability; Effectiveness/cost-effectiveness; Acceptability; Side effects/safety; Equity) criteria guided the selection of BCT delivery formats.

**Results:**

Formats for delivering the 11 BCTs were agreed upon, for example, a paper medicines diary was selected to deliver the BCT ‘Self-monitoring of behaviour’. To help tailor the intervention, BCTs were categorised into ‘Core’ and ‘Optional’ BCTs. For example, ‘Feedback on behaviour’ and ‘Action planning’ were selected as ‘Core’ BCTs (delivered to all patients), whereas ‘Prompts and cues’ and ‘Health consequences’ were selected as ‘Optional’ BCTs. A paper-based adherence assessment tool was designed to guide intervention tailoring by mapping from identified adherence problems to BCTs. The intervention was designed for delivery over three appointments in the pharmacy including an adherence assessment at Appointment 1 and BCT delivery at Appointments 2 and 3.

**Conclusions:**

This paper details key decision-making processes involved in moving from a list of BCTs through to a complex intervention package which aims to improve older patients’ medication adherence. A novel approach to tailoring the content of a complex adherence intervention using ‘Core’ and ‘Optional’ BCT categories is also presented. The intervention is now ready for testing in a feasibility study with community pharmacists and patients to refine the content. It is hoped that this detailed report of the intervention content/design process will allow others to better interpret the future findings of this work.

## Background

Medication non-adherence is a major challenge affecting healthcare systems globally and older adults, prescribed multiple medications, are seen as a high priority group for intervention [1–3]. Previous complex interventions designed to address non-adherence have shown only limited effectiveness, which may be attributable to poor design and lack of a theoretical underpinning [1, 4–6]. To improve effectiveness, the UK Medical Research Council (MRC) recommend the use of a systematic approach encompassing: (1) Development; (2) Feasibility and pilot testing; (3) Evaluation; and (4) Implementation [7].

Whilst there has been a substantial increase in the number of published studies that report on the feasibility and pilot testing of complex interventions in recent years, fewer publications have focused on intervention development and design processes [8, 9]. Intervention development has previously been compared to a “black box”, given that key factors such as decision-making regarding intervention design take place in this phase but are rarely reported in any great detail [9]. Where development decisions are reported, these are often combined with feasibility study findings, thus limiting the amount of information provided. To avoid wasting research resources, including time and money, more attention needs to be paid to reporting key development decisions. This will help to inform others who may wish to replicate, or build upon, the basis of intervention design. This has led to the call for researchers to publish intervention development studies which have been defined by Hoddinott [9] as *“A study that describes the rationale, decision making processes, methods and findings which occur between the idea or inception of an intervention until it is ready for formal feasibility, pilot or efficacy testing prior to a full trial or evaluation.”* Through the increased reporting of these types of studies in the literature, other researchers can learn from both successful and unsuccessful approaches and add to the scientific rigour of complex intervention research.

The MRC’s complex intervention guidance recommends that researchers incorporate theory into the intervention design process to aid understanding of its mechanism of action [7]. However, a recent systematic review has shown that psychological theory is rarely used to guide the selection of intervention components to include in adherence interventions delivered to older adults prescribed multiple medicines [10]. Accordingly, research was conducted that used the Theoretical Domains Framework (TDF) of behaviour change to identify key targets for changing older adults’ adherence. This process is described in detail in a related publication [11]. In summary, eight theoretical domains (e.g. motivation and goals) were identified as key targets and mapped to 11 BCTs (e.g. action planning) that could be delivered in an intervention package to bring about behaviour change. In this context, it was identified that the intervention and BCTs would need to be tailored, at an individual-level, to address the underlying reasons for non-adherence which often varied between patients. Community pharmacists were selected as the provider for this type of adherence support intervention due to their accessibility, frequent contact with patients and recent evidence that supports pharmacists’ involvement in improving medicines use by patients [1, 12].

Following identification of suitable BCTs for the target behaviour, the next stage of intervention development is to operationalise each of these and combine them into an intervention package, prior to future feasibility and pilot testing [7]. Although an important step, there is limited guidance in the literature on exactly how to operationalise and combine multiple BCTs into an intervention package that could potentially be delivered in the intended setting. In addition, it is unclear how such interventions should be tailored to each patient’s needs. In order for a tailored intervention to be replicable, the process needs to be systematic and reported in detail in any published reports. Accordingly, the current paper reports on how 11 BCTs derived from previous qualitative research with older patients [11] were operationalised and combined into a complex intervention [IDentification of Medication Adherence Problems (ID-MAP) intervention] that can be tailored by community pharmacists, as part of a future feasibility study.

## Methods

**Fig. 1:**
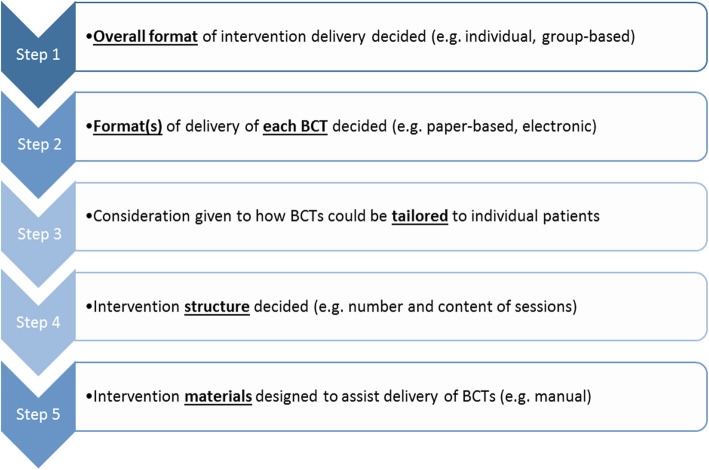
Key stages involved in designing an intervention package to improve medication adherence in older patients in the community pharmacy setting

Figure 1 outlines the key stages that were undertaken to develop a complex theory-based intervention package that would be suitable for future feasibility testing in community pharmacies with pharmacists and older patients in the next phase of the research programme. These stages are described in further detail in the subsequent text. The intervention content has been reported in line with the TIDieR (Template for Intervention Description and Replication) guidelines [13] (see completed checklist in Supplementary File 1).

### Step 1: selection of overall format of delivery

As alluded to previously, it was decided at the outset of the project that this type of adherence support intervention would be designed for delivery by community pharmacists in the primary care setting. This decision was made based on two recent Cochrane reviews that support pharmacists’ involvement in interventions to improve patients’ use of medications [1, 12]. In addition, the accessibility of community pharmacists and frequency of contacts with patients (e.g. when collecting repeat prescriptions) provides opportunities to support adherence on an ongoing basis [11]. The target audience for receipt of the intervention is older patients (65 years or older) who are prescribed four or more regular medications (polypharmacy). The intervention has not been designed for patients with cognitive impairment (i.e. with a confirmed diagnosis of dementia) as these patients were not included in the original focus group study which formed the theoretical basis of the intervention [11]. Furthermore, patients with cognitive impairment may have additional support needs that extend beyond the scope of the current research project. Further decisions regarding the overall format (e.g. remote verses face-to-face delivery) were made based on research team members’ experience of developing interventions for the target audience [13, 14] and knowledge of the selected setting.

### Step 2: selection of format for the delivery of each BCT

Once the overall format of intervention delivery was decided, the next step was to consider the format for delivering each BCT. Potential delivery formats for BCTs considered included verbal delivery, delivery through written materials (e.g. leaflets) or through technology-based solutions (e.g. wearable devices/smart phones). These formats may also have incorporated images, videos and/or audio.

Definitions for each BCT have been provided in the BCT taxonomy Version 1.0 (BCTTv1) which acted as a useful starting point for discussing potential formats of delivery [15]. For example, the BCT ‘Self-monitoring of the behaviour’ has been defined as a way to, *‘Establish a method for the person to monitor and record their behavior(s) as part of a behavior change strategy’.* Potential delivery formats identified for delivering this BCT included: (1) printed materials (e.g. providing patients with a written medicines diary) or (2) provision of digital technology to record medication use (e.g. a mobile telephone application). Although reports from the UK communications regulator Ofcom have shown that an increasing proportion of older patients in the UK now have access to technology such as smartphones (between 2015 and 2016 39% of adults aged 65–74 and 15% of over 75 s had access to a smartphone), the vast majority still do not have access. This was therefore taken into consideration when making decisions about the delivery of individual BCTs [16].

Prior to conducting feasibility testing with the target audience, the research team made decisions based on the likely suitability of the proposed format for delivering each of the 11 BCTs. The selected setting, target audience, target behaviour and findings from previous research [11] were taken into consideration and the APEASE criteria (Affordability; Practicability; Effectiveness/cost-effectiveness; Acceptability; Side effects/safety; Equity), developed by Michie et al. [17], were used to systematically guide this decision-making process (see Table 1). Decisions in the current study were informed by considering four out of six APEASE criteria: ‘Affordability’, ‘Practicability’, ‘Acceptability’, ‘Equity’. Due to insufficient evidence available on the safety and effectiveness/cost-effectiveness of individual BCTs (in the context of improving older patients’ medication adherence) the ‘effectiveness-cost-effectiveness’ and ‘safety-side effects’ APEASE criteria were not applied in the current study.

**Table 1:** APEASE criteria (adapted from [17])

**APEASE criterion**	**Description**	**Considerations for the target setting and audience**
**A**ffordability	Costs associated with the design and delivery of each proposed BCT delivery format should be considered by intervention designers.	Due to the financial constraints associated with research and community pharmacies, key costs, such as those associated with technology were considered.
**P**racticability	The practicality of the design and delivery processes for each proposed BCT delivery format should be considered by intervention designers.	The practicality of preparing and delivering the BCTs via each proposed format in community pharmacies was considered (e.g. the time and input required from the research team and community pharmacists).
**E**ffectiveness/cost-effectiveness	Intervention designers should consider the cost-effectiveness and effectiveness of delivering the BCT using each proposed format, where sufficient evidence is available.	This criterion was not applied in the current study due to the lack of evidence available on the effectiveness of each BCT in relation to improving adherence in older adults.
**A**cceptability	The likely acceptability of each proposed BCT delivery format from the view point of intervention recipients and providers should be considered by intervention designers.	The likely acceptability of delivery formats from the viewpoints of patients and community pharmacists were considered (e.g. potential time required to complete documentation).
**S**ide effects/safety	The potential for side effects or safety issues associated with BCT delivery formats should be considered by intervention designers.	This criterion was not applied in the current study due to the lack of evidence currently available on the safety of each BCT in relation to improving adherence in older adults.
**E**quity	The potential reach of the intervention to disadvantaged groups should be considered by intervention designers when selecting BCT delivery formats.	The likely reach of the intervention to disadvantaged groups of older patients (e.g. those with low literacy levels) was considered.

### Step 3: tailoring BCTs (identifying ‘Core’ and ‘optional’ BCTs)

Findings from our previous study highlighted the wide range of underlying reasons for non-adherence in older patients. It would therefore be unnecessary and potentially inappropriate to deliver all 11 BCTs to each individual patient [11]. Therefore, the research team recognised the importance of adopting a tailored approach to BCT delivery, taking into account each patient’s individual needs. There is a lack of guidance in the literature for researchers involved in designing interventions, on how best to approach this tailoring process. However, the process of assigning BCTs as ‘Core’ and ‘Optional’ components of the intervention, as outlined in the current study, is a potential approach that could facilitate this. A group consensus approach (involving discussions amongst members of the research team: DP, CH, CR) and with reference to preceding research [11] were employed to select BCTs that may be useful for all patients, regardless of the underlying reasons that explain why they are non-adherent– these BCTs were categorised as ‘Core’ BCTs. BCTs that were deemed to be useful only under certain circumstances, depending on each patient’s needs, were categorised as ‘Optional’ BCTs. For example, an unintentionally non-adherent patient, such as a patient who forgets, would require different types of adherence solutions (e.g. BCT: prompts/cues), compared with a patient who is intentionally non-adherent, for example, due to incorrect beliefs about side effects (e.g. BCT: health consequences).

### Step 4: intervention structure

Following the categorisation of ‘Core’ and ‘Optional’ BCTs, these were combined and structured as part of an intervention package. The research team recognised that it would not be feasible to deliver all required BCTs at one appointment, for example, the BCT ‘Review of behaviour goal’ can only be delivered once a period of time has elapsed since delivery of the BCT ‘Goal setting-behaviour’. This is necessary to give the patient time to implement the agreed behavioural goal before reviewing this. Consequently, the number of appointments and time period between appointments was decided based upon: the minimum number of appointments necessary to deliver BCTs and conduct follow-up with patients (minimum of two), background knowledge of the proposed delivery setting (e.g. current time constraints in community pharmacies which restrict appointment length) and the previous experience of research team members’ who have been involved in developing similar interventions [13, 14]. Consideration was also given to categorising BCTs into groups as it was recognised that pharmacists may not be familiar with the terms ‘BCT’ or ‘behaviour change technique’ as these stem from the field of psychology. Therefore, the 11 BCTs in the intervention were separated into four adherence solution categories (see results for further details).

### Step 5: design of intervention materials and training package

To support BCT delivery by community pharmacists, a variety of materials and resources were developed including: an intervention manual, a paper-based intervention record (which contained an adherence assessment tool to guide BCT tailoring and provided space to record notes on BCT delivery), supporting materials (e.g. patient leaflets) and a brief face-to-face training package. The intervention record has been provided in Supplementary File 2. Other materials are available from the authors on request. For complex interventions, an intervention manual and/or training package that outlines exactly how the intervention should be delivered can help to ensure consistency in delivery by intervention providers and improve intervention fidelity (i.e. the extent to which the intervention is delivered as intended) [18]. The development of intervention materials and resources was based on: findings from a previously conducted systematic review [10] and other relevant literature [1, 4], findings from the prior qualitative research [11], research team members’ previous experience with developing interventions for delivery by pharmacists and in community pharmacies [13, 14] and knowledge of the proposed delivery setting. All intervention materials and resources underwent an extensive review process within the research team prior to external review and testing by three pharmacists from the wider research group who were not involved in the project (at the time of conducting the study, two of the pharmacists were working within community pharmacies on a part-time basis).

## Results

The results from each of the five steps involved in this intervention development study (as outlined in Fig. 1) are presented below.

### Results from step 1 (overall format of delivery)

Face-to-face delivery was selected for this community pharmacy-based intervention, as opposed to remote delivery (e.g. via telephone, video call). Similar interventions (services) currently offered in the community pharmacies in Northern Ireland, such as the Medicines Use Review (MUR) service [19], are delivered face-to-face and so this was deemed likely to be acceptable to both pharmacists and patients and support future implementation into practice [20].

### Results from step 2 (individual BCT delivery formats)

From the definitions of BCTs noted in BCTTv1 [15] and process of applying the subset of APEASE criteria: ‘Affordability’, ‘Practicability’, ‘Acceptability’, and ‘Equity’ [17], consensus was reached on the formats for the delivery of each of the 11 BCTs. The results from this decision-making process are presented in Table 2.

**Table 2:** Selected formats for delivering behaviour change technique (BCTs) as part of a community pharmacy-based intervention

**BCT**	**Definition provided in BCT Taxonomy Version 1 (BCCTTv1) **[15]	**Potential formats for delivering the BCT**	**Final selected format(s) for delivering the BCT (including reasons)**
Health consequences	*‘Provide information (*e.g. *written, verbal, visual) about health consequences of performing the behaviour’*	**Option 1:** Provision of verbal information by pharmacists **Option 2:** Provision of printed and/or written information by pharmacists	**Options 1 and 2:** These were deemed practical to deliver in community pharmacies. Based on findings from previous focus groups, these formats were deemed likely to be well accepted by patients. These options are both low-cost and giving patients a choice would aim to overcome potential equity issues (e.g. poor literacy).
Restructuring the physical environment	*‘Change or advise to change the physical environment in order to facilitate performance or create barriers to the unwanted behaviours (other than prompts/cues, rewards and punishment)’*	**Option 1:** Verbal record of agreed changes **Option 2:** Written instructions of agreed changes	
Social support (unspecified)	*‘Advise on, arrange or provide social support (*e.g. *from friends, relatives, colleagues, ‘buddies’ or staff) or non-contingent praise or reward for performance of the behaviour. It includes encouragement and counselling but only when it is directed at the behaviour’*	**Option 1:** Verbal record outlining a social support plan **Option 2:** Written instructions outlining the social support plan	
Feedback on behaviour	*‘Monitor and provide information or evaluative feedback on performance of the behaviour (*e.g. *form, frequency, duration, intensity)’*	**Option 1:** Provision of verbal information by the pharmacist **Option 2:** Provision of printed/written information by the pharmacist	**Option 1:** This was considered to be low-cost and practical to deliver. This format was also deemed likely to be accepted by pharmacists/patients. Option 2 was not chosen as it could be time-consuming to prepare written/printed feedback for each patient given the time constraints in community pharmacies. The selected format will also aim to overcome equity issues (e.g. poor literacy).
Prompts/ Cues	*‘Introduce or define environmental or social stimulus with the purpose of prompting or cueing the behaviour. The prompt or cue would normally occur at the time or place of the performance’*	**Option 1:** Reminder stickers **Option 2:** Visual or situational prompts (e.g. location, time) **Option 3:** Electronic devices (e.g. Reminder app/smartwatches)	**Options 1 and 2:** These were deemed to be low-cost, likely to be accepted by patients/pharmacists and practical to recommend in community pharmacies. Option 3 was not chosen because of the high costs associated with providing electronic devices to patients, potential reach to patients without access to devices and acceptability to older patients.
Self-monitoring of the behaviour	*‘Establish a method for the person to monitor and record their behaviour(s) as part of a behaviour change strategy’*	**Option 1:** Paper medicines diary **Option 2** Electronic device (e.g. mobile phone app)	**Option 1**: This was selected due to the low-costs involved in development, practicality of providing this in community pharmacies and likely acceptability to older patients. Option 2 was not chosen due to the high costs associated with designing an app and provision of devices, as well as user-training requirements, and potential equity issues (e.g. reach to patients without mobile phone access).
Goal setting-behaviour	*‘Set or agree a goal defined in terms of the behaviour to be achieved’*	**Option 1:** Verbal agreement of goal(s) **Option 2:** Written record of agreed goal(s)	**Options 1 and 2**: These were selected as both may be required, depending on each patient’s circumstances. Both options were considered low-cost, likely to be acceptable to patients and pharmacists and practical to deliver in the proposed setting. The inclusion of both formats would also aim to overcome equity issues (e.g. poor literacy).
Goal setting-outcome	*‘Set or agree a goal defined in terms of a positive outcome of wanted behaviour’*		
Review of behaviour goal	*‘Review behaviour goal(s) jointly with the person and consider modifying goal(s) or behaviour change strategy in light of achievement. This may lead to re-setting the same goal, a small change in that goal or setting a new goal instead of (or in addition to) the first, or no change.’*	**Option 1:** Verbal review of goal(s) **Option 2**: Written record of goal review discussion	
Review of outcome goal	*‘Review outcome goal(s) jointly with the person and consider modifying goal(s) in light of achievement. This may lead to re-setting the same goal, a small change in that goal or setting a new goal instead of, on in addition to the first’*		
Action planning	*‘Prompt detailed planning of performance of the behaviour (must include at least one of context, frequency, duration and intensity). Context may be environmental (physical or social) or internal (physical, emotional or cognitive’*	**Option 1:** Verbal agreement of an action plan **Option 2:** Written record of agreed action plan	

### Results from step 3 (tailoring BCTs: ‘Core’ and ‘optional’ BCTs)

Five BCTs (out of 11) were considered to be potentially relevant for all non-adherent patients (irrespective of the underlying reasons for this). Subsequently these BCTs were selected as key intervention components that would be delivered to all non-adherent patients and termed ‘Core’ BCTs (see Table 3). Six BCTs were categorised as ‘Optional’ BCTs as they were considered unlikely to be relevant for all patients. Alternatively, these ‘Optional’ BCTs could be delivered following an assessment of each individual’s underlying reasons for medication non-adherence (see Table 3).

**Table 3:** ‘Core’ and ‘Optional’ BCTs in the intervention package

**BCT**	**‘Core**^**1**^**’ or ‘Optional**^**2**^’	**Brief description of how the BCT will be delivered as part of the intervention in a future feasibility study**
Self-monitoring of the behaviour	Core	A paper medicines diary will be offered to all patients.
Goal-setting (behaviour)	Core	A goal focusing on improving medication use will be set by the patient and pharmacist. The goal could then be reviewed at a follow-up appointment.
Review of behaviour goal	Core	
Action planning	Core	A detailed plan of how the patient will perform the behaviour will be jointly developed (e.g. including specific times).
Feedback on behaviour	Core	Feedback will be given to each patient following a review of their medicines diary (e.g. patterns of missed doses).
Health consequences	Optional	Information on the health consequences of adherence/non-adherence will be given to patients who are intentionally non-adherent.
Social support (unspecified)	Optional	A verbal or written plan for obtaining support from others (e.g. family, pharmacy staff) could be developed for those who require this. For example, pharmacy staff may support with the patient with the synchronisation of medication supplies.^3^
Prompts and cues	Optional	A social or environmental stimulus that cues or acts as a prompt could be recommended to patients who forget to take medications.
Restructuring the physical environment	Optional	A change to the physical environment could be recommended for patients who experience practical difficulties (e.g. changes to packaging, Monitored Dosage Systems).
Goal setting (outcome)	Optional	Goals focusing on the positive outcomes of taking medications (e.g. symptom reduction) could be set by patients deemed to have low motivation. The goal could then be reviewed at the follow-up appointment.
Review of behaviour goal	Optional	

^*1*^*BCTs that were selected as potentially suitable for delivery to all patients;*^*2*^*BCTs that were selected as suitable for delivery only when deemed necessary by pharmacists based on an adherence assessment of underlying reasons for non-adherence.;*^*3*^*The synchronisation of medication supplies ensures that patients are dispensed all regular medications on the same day which helps to avoid multiple visits to the pharmacy*

### Results from step 4 (intervention structure)

To guide the structure of the intervention, the research team considered that most services (i.e. interventions) currently delivered by pharmacists in the community setting in Northern Ireland (NI) are based on one or two appointments. In addition, following the definitions provided in BCTTv1 [15], it was noted that some BCTs could only be delivered following the implementation of another BCT. For example, in this context the BCT ‘Feedback on behaviour’ (Core BCT) could only be delivered following delivery and implementation of the BCT ‘Self-monitoring of the behaviour’. Therefore, to facilitate this delivery of BCTs, a two-appointment model was initially considered for this intervention. However, it was noted during discussions amongst the research team that pharmacists may require additional time to prepare resources (e.g. medicines diary) required to deliver BCTs and to liaise with other members of the healthcare team [e.g. General Practitioner (GP)] for this type of behaviour change intervention. Consequently, the intervention was designed for delivery over three face-to-face appointments, with Appointment 1 focusing on assessment and Appointments 2 and 3 focusing on BCT delivery and follow-up, respectively. Table 4 outlines the activities that will be delivered at each appointment of the intervention in a future feasibility study.

**Table 4:** Behaviour change techniques (BCTs) that will be delivered at each appointment of the intervention

**Appointment number**	**Activity**	
Appointment 1	An assessment of the underlying reasons/causes of non-adherence (guided by a paper-based adherence assessment tool)	
Appointment 2	**‘Core’ BCTs** • ‘Self-monitoring of the behaviour’ • ‘Goal-setting (behaviour)’ • ‘Action planning’	**‘Optional’ BCTs** • ‘Restructuring the physical environment’ • ‘Prompt/cues’ • ‘Social support (unspecified)’ • ‘Health consequences’ • ‘Goal setting (outcome)’
Appointment 3	**‘Core’ BCTs** • ‘Feedback on behaviour’ • ‘Review of behaviour goal’	**‘Optional’ BCT** • ‘Review of outcome goal’

Key: BCTs = Behaviour change techniques

In a future feasibility study, Appointment 2 will be scheduled to take place one to 2 weeks following Appointment 1 to allow adequate time for the preparation of resources and contact with other healthcare professionals (e.g. the patient’s GP) if required. GPs will be contacted at the outset of the future study and given the option to receive written communication forms during the study with information on the outcomes of the adherence assessment for their patients. This will clearly indicate the whether any action is required on behalf of the GP or if it is solely for information purposes.

A follow-up review appointment (Appointment 3) will then be recommended after a minimum of 4 weeks to allow sufficient time for the patient to implement agreed BCTs (i.e. solutions). At the final appointment, pharmacists and patients will then discuss any BCTs that were recommended/delivered at the previous appointment (e.g. medicines diary). The intervention will therefore be delivered to patients over a period of five to eight-weeks. As this is a complex patient group prescribed multiple medications for a range of clinical conditions, multiple and complex adherence issues may arise. Due to the time restrictions in the community pharmacy setting issues identified may have to be addressed over multiple sessions. Pharmacists should prioritise the most significant problems where the clinical implications of non-adherence would be greater, for example, non-adherence to insulin or anti-epileptics. When prioritising issues pharmacists should also consider what is going to have the most impact and take into consideration the patient’s viewpoint on what is most important to them.

**Fig. 2:**
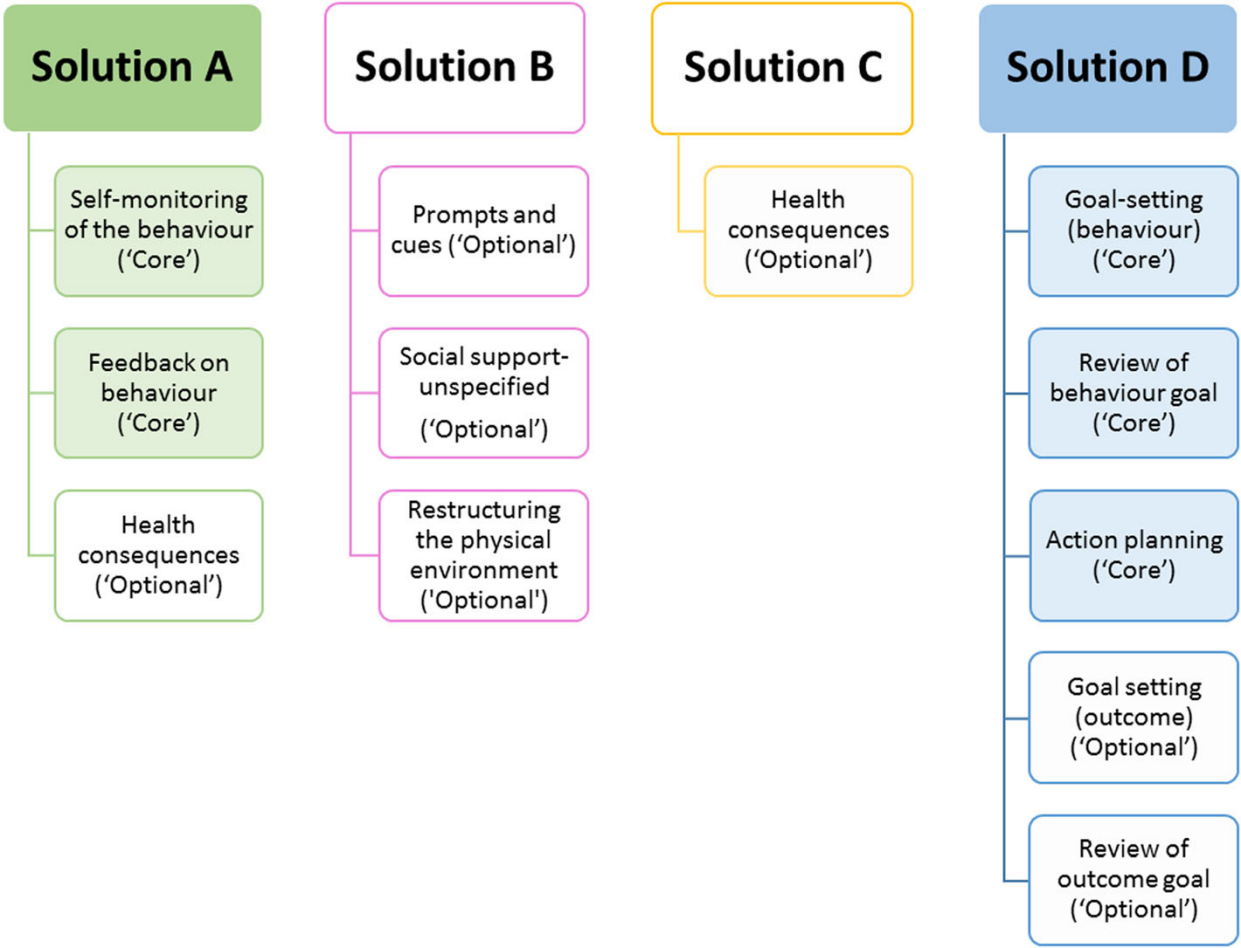
Structuring of ‘Core’ and ‘Optional’ BCTs into adherence solution categories

As stated previously, to help structure the intervention and avoid the term ‘Behaviour Change Technique (BCT)’, the 11 BCTs in the intervention were divided into four categories (Solution A, B, C and D) - these are shown in Fig. 2.

Findings from previous research, which included focus groups with older patients [11], provided key contextual information that informed the operationalisation of BCTs in the current intervention. For example, focus group participants reported that they found the medication lists they received on discharge from hospital to be beneficial. Therefore, when operationalising the BCT ‘Self-monitoring of the behaviour’ a similar format was used in the design of the medicines diary. Participants in the focus groups also reported locations that they found useful for storing medications as these acted as visual prompts (e.g. bed-side table, kitchen counter top) – this information guided decisions around the types of reminder strategies (BCT: prompts/cues) selected for inclusion in the current intervention.

### Results from step 5 (intervention materials and training package)

A paper-based intervention booklet has been developed to guide intervention appointments and facilitate the recording of notes (See Supplementary File 2). This intervention booklet contains the following four sections: (1) medication history (completed pre-appointment 1); (2) adherence assessment tool (see below) (completed at Appointment 1); (3) adherence solutions (completed at Appointment 2); (4) review of adherence solutions (completed at Appointment 3).

The adherence assessment tool has been developed to guide pharmacists in selecting ‘Optional’ BCTs to deliver to individual patients, in addition to the ‘Core’ BCTs that will be delivered to all patients. This tool, which has been included in Section 2 of the intervention record, has seven open style questions (including additional prompts) to support pharmacists in identifying the underlying reasons for medication non-adherence during consultations with patients. These questions have been developed to facilitate identification of problems related to: patients’ knowledge of medications (Q1), organisational or routine-related barriers (Q2), physical or practical barriers (Q3), social support requirements (Q4), forgetting (Q5), intentional reasons for non-adherence (Q6) and patients’ motivation (Q7). The questions have been developed based on the findings from the previous qualitative research and together, these explore the seven key theoretical (TDF) domains that were identified as key intervention targets for changing older patients’ adherence behaviour [11]. For example, Question 4 explores barriers and facilitators (identified in previous research) within the ‘Social influences’ domain in the TDF and Questions 5 and 6 cover the ‘Memory, attention and decision-making processes’ domain. Using this tool, pharmacists will be able to select any relevant ‘adherence problems’ that they identify during discussions with the patient at Appointment 1 (by ticking the relevant box). Each ‘adherence problem’ will then be linked to at least one suggested adherence solution (i.e. BCT) which can be recommended/delivered by the pharmacist at Appointment 2.

Other paper-based materials that have been developed to assist pharmacists in delivering the 11 BCTs in a future feasibility study include: a paper-based medicines diary (BCT: Self-monitoring of the behaviour); leaflets on voicing concerns about medications and generic medications (BCT: Health consequences), goals and action planning activity sheet (BCTs: goal-setting BCTs and action planning) and reminder stickers (BCT: prompts and cues).

A brief training session was also developed as a starting point for exploring the intensity and type of training required by pharmacists to enable them to deliver this type of tailored adherence intervention in practice. The training, which will be delivered as part of a future feasibility study, will include a didactic Microsoft PowerPoint® presentation covering background information on non-adherence and how to deliver the intervention in the context of the community pharmacy setting. Step-by-step information, pictures/copies of intervention materials and illustrative examples will be used as part of the training to help engage pharmacists. The developed training session has undergone external review by the three pharmacists mentioned previously. All of the information provided in the training package has also been covered in greater detail in a paper-based intervention manual (available from the authors upon request). Both the training package and intervention manual aim to train pharmacists on identifying adherence problems, recommending tailored solutions (i.e. BCTs) and reviewing these solutions over three appointments in the pharmacy. In addition, two illustrative patient scenarios of varying complexity have been developed based on real life scenarios that were discussed in the prior patient focus groups [11].

## Discussion

This paper presents a complex intervention package that has been designed to incorporate 11 BCTs that were identified as potential ‘active intervention ingredients’ to improve older patients’ adherence to multiple medications [11]. This paper has described the key decision-making processes involved in moving from a list of BCTs (and definitions provided in BCTTv1) through to the final intervention package. This type of intervention design and development work is rarely reported in sufficient detail in published reports. It is therefore hoped that this report will allow others to better interpret and build on the future findings of this work.

It has been suggested by Hoddinott [9] that developing complex interventions requires a delicate balance between science and creativity. In this context, due to the time pressures faced in community pharmacies, creativity is required to ensure that any developed intervention is likely to be feasible for pharmacists to implement, whilst still containing the proposed active ingredients (BCTs) which are hypothesised to bring about behaviour change. The APEASE criteria [17] proved useful in this context in terms of guiding decision-making at this early stage of intervention development by aiding the selection of the most suitable BCT delivery formats to proceed to feasibility testing within the target setting and in the context of the target audience of older patients. It should be noted that the selected formats for BCT delivery formats may have differed if the intervention was designed to target a different age group or patients with cognitive impairment. For example, if the intervention was designed to target patients aged 18–65 years, then technology-based solutions such as an electronic medication diary for delivery of the BCT ‘Self-monitoring’ may have been selected instead on a paper-based version.

A major design consideration for this intervention was exactly how it could be tailored, in a systematic way, to each older patient’s needs. This was deemed important given that previous research indicated that older patients prescribed polypharmacy were often non-adherent for a range of different reasons which can vary both between and within patients (e.g. different reasons for different medications) [11]. It was agreed that the delivery of all intervention components (i.e. BCTs) to all patients would be a time-consuming and inefficient approach and may be one of the reasons why a large number of previously tested interventions have failed to lead to large improvements in adherence. This is one of the first studies to report exactly how theory-based BCTs will be tailored to target the key underlying reasons for each patient’s non-adherence. The paper-based adherence assessment tool that has been developed and reported in this paper seeks to facilitate pharmacists in tailoring adherence solutions (i.e. BCTs) in a systematic way— this approach aims to help ensure consistency across providers in future feasibility and evaluation studies of the intervention. Research which explored medication adherence in adults (18 years+) with cardiovascular disease, led to the development of a 30-item questionnaire for patients to complete to help identify the underlying reasons for medication non-adherence. This was subsequently refined to a 10-item version as the previous version was deemed to be too lengthy for practice [21, 22]. For older patients, who were the focus of the current project, a conversational style approach was deemed most suitable to aid identification of the underlying reasons of non-adherence. This decision was made based on findings from the previous qualitative research [11], whereby patients emphasised the importance of conversations with healthcare professionals in establishing/maintaining relationships and addressing any medication-related problems they faced.

### Strengths and limitations

A strength of this study is that the intervention has been designed in line with the MRC complex intervention development framework [7] and the contents of the intervention have been reported as recommended by the TIDieR guidelines (Supplementary File 1). These guidelines advocate for clearer reporting of interventions so that they can be easily interpreted and replicated by others [23]. This paper has reported on important decisions that were made in designing the complex intervention package. This decision-making process, which involved a group consensus approach, was challenging, however, the APEASE criteria were useful in both guiding this process and facilitating reporting of the findings [17].

The current study adds to the literature by describing exactly how a complex adherence intervention was designed so that it could be tailored at an individual-level. The need for tailored interventions (also referred to as ‘personalised’ interventions) has become an area of great interest in the field of adherence research as it is thought that this approach may help to improve intervention effectiveness [24]. Tailoring of adherence interventions in the literature is, however, currently uncommon—this has been illustrated in a recent meta-analysis of 771 adherence interventions delivered to adult patients (18 years+) which found only nine studies that reported individual-level tailoring [6]. Keogh et al. [25] have also recently advocated for the inclusion of ‘core’ and ‘optional’ BCTs in complex interventions, following their recent feasibility study that found that a physiotherapist-led self-management group intervention containing a large number of BCTS (*n* = 31) was delivered with low to moderate fidelity. This type of tailoring could be particularly useful in contexts where there are high levels of heterogeneity amongst patients and in busy healthcare environments where time restrictions limit the duration of sessions/appointments.

The development work reported here has involved extensive review of the intervention materials within the research team and by external pharmacists who were practising in the community setting. Although findings from patient-focused research (focus groups) carried out prior to this study informed many of the decisions made regarding intervention design [11], due to time restrictions and funding constraints, patients were not directly involved in the final decisions made regarding intervention design at this stage. Nonetheless, the next stage of this research will involve testing the proposed complex intervention package in the selected setting with the target audience to help refine the intervention design and content. This will involve seeking feedback from older patients prescribed multiple medications, as well as community pharmacists.

## Conclusions

This study reports on how a theory and evidence-based intervention has been designed for delivery by community pharmacists to older patients to improve adherence to multiple medications. It details how the intervention package was designed to incorporate 11 theory-based BCTs identified from prior research and the key decision-making processes involved with this difficult approach. The complex intervention package reported here will now undergo feasibility testing in community pharmacies with the target audience—pharmacists and patients. This will help to explore whether refinements are required to ensure the intervention is both useful and acceptable to patients and pharmacists, before proceeding to pilot and larger scale evaluation testing.

## Supplementary information

**Additional file 1: Supplementary File 1.** TIDieR (Template for Intervention Description and Replication) Checklist.

**Additional file 2.** Intervention record.

## Data Availability

The dataset(s) supporting the conclusions of this article is (are) included within the article [and its supplementary file(s)] and/or are available from the corresponding author upon reasonable request.

## Abbreviations

BCT: Behaviour change technique
BCTTv1: Behaviour change technique taxonomy- Version 1
MRC: Medical Research Council
MUR: Medicine Use Review service
NI: Northern Ireland
TDF: Theoretical Domains Framework
TIDieR: Template for Intervention Description and Replication
