# Supplementary material for: Development of a complex community pharmacy intervention package using theory-based behaviour change techniques to improve older adults’ medication adherence
Source: BMC Health Serv Res. 2020 May 13;20:418. doi: 10.1186/s12913-020-05282-7 (PMC7222450; doi:10.1186/s12913-020-05282-7)
Supplement: Supplementary file 1 — Additional file 1: Supplementary File 1. TIDieR (Template for Intervention Description and Replication) Checklist. [file 12913_2020_5282_MOESM1_ESM.docx]

# Supplementary File 1: TIDieR (Template for Intervention Description and Replication) Checklist

| **TIDieR item number** | **Description of item** | **Location where relevant information can be found** |
| --- | --- | --- |
| **1.** | **BRIEF NAME**  Provide the name or a phrase that describes the intervention. | Title/Page 4 |
| **2.** | **WHY**  Describe any rationale, theory, or goal of the elements essential to the intervention. | Page 4-5 |
| **3.**  **4.** | **WHAT**  **Materials:** Describe any physical or informational materials used in the intervention, including those provided to participants or used in intervention delivery or in training of intervention providers. Provide information on where the materials can be accessed (e.g. online appendix, URL).  **Procedures:** Describe each of the procedures, activities, and/or processes used in the intervention, including any enabling or support activities. | Page 9, Supplementary File 2 and upon request from authors  Pages 7-9, Tables 3 and 4, Figure 2 |
| **5.** | **WHO PROVIDED**  For each category of intervention provider (e.g. psychologist, nursing assistant), describe their expertise, background and any specific training given. | Page 6 |
| **6.** | **HOW**  Describe the modes of delivery (e.g. face-to-face or by some other mechanism, such as internet or telephone) of the intervention and whether it was provided individually or in a group. | Pages 6-7 & 10 |
| **7.** | **WHERE**  Describe the type(s) of location(s) where the intervention occurred, including any necessary infrastructure or relevant features. | Page 9 |
| **8.** | **WHEN and HOW MUCH**  Describe the number of times the intervention was delivered and over what period of time including the number of sessions, their schedule, and their duration, intensity or dose. | Table 4, Page 10-11 |
| **9.** | **TAILORING**  If the intervention was planned to be personalised, titrated or adapted, then describe what, why, when, and how. | Pages 7-8 and 10, Table 3 |
| **10.** | **MODIFICATIONS**  If the intervention was modified during the course of the study, describe the changes (what, why, when, and how). | Not applicable |
| **11.**  **12.** | **HOW WELL**  Planned: If intervention adherence or fidelity was assessed, describe how and by whom, and if any strategies were used to maintain or improve fidelity, describe them.  Actual: If intervention adherence or fidelity was assessed, describe the extent to which the intervention was delivered as planned. | Not applicable  Not applicable |
