# Supplementary material for: Development of a complex community pharmacy intervention package using theory-based behaviour change techniques to improve older adults’ medication adherence
Source: BMC Health Serv Res. 2020 May 13;20:418. doi: 10.1186/s12913-020-05282-7 (PMC7222450; doi:10.1186/s12913-020-05282-7)
Supplement: Supplementary file 2 — Additional file 2. Intervention record. [file 12913_2020_5282_MOESM2_ESM.pdf]

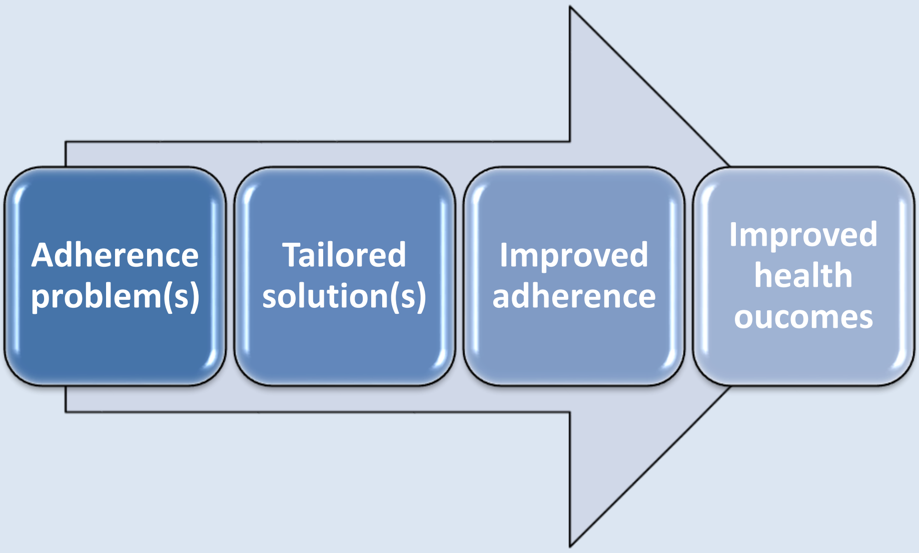

ID-MAP Booklet

Please complete one booklet per patient

Pharmacy ID:

Patient ID:

IMPORTANT

Ensure you have completed all aspects of screening and recruitment before proceeding with Appointment 1

Consent Form 2 completed *(please tick)* ☐

Health-related quality of life questionnaire completed *(please tick)* ☐

Please complete appointment information on page 2 at the START and END of each appointment

Contents

Appointment information……………………………………………………………………………………………………….2
Section 1: Medication Details ……………………………………………………………………………………………….3
Section 2: ID-MAP Tool ………………………………………………………………………………………………………. 4-8
Appointment 1 Checklist ……………………………………………………………………………………………………….8
Section 3: Solution Summary……………………………………………………………………………………………. 9-11
Appointment 2 Checklist…………………………………………………………………………………………………….12
Section 4: Review of solutions/feedback…………………………………………………………………………….13
Appointment 3 Checklist……………………………………………………………………………………………………..14

Section 1
- Complete medication details as far as possible **BEFORE Appointment 1**
- Confirm medication details **DURING Appointment 1**

Section 2
- Ask all questions in the ID-MAP Tool **DURING Appointment 1** to identify adherence problems (Appointment 1 ends here)
- Map adherence problems to adherence solutions **BEFORE Appointment 2**

Section 3
- Prepare adherence solutions and make planning notes on the Solution Summary **BEFORE Appointment 2**
- Deliver adherence solutions **DURING Appointment 2** (Appointment 2 ends here)

Section 4
- Complete review of adherence solutions and give feedback **DURING Appointment 3** (end of intervention)

Reminder instructions for pharmacists

- This booklet will help guide your discussions with the patient for each appointment of the ID-MAP Intervention.
- Please refer to the intervention manual for detailed instructions on how to complete this booklet.
- Brief reminder instructions are provided at the top of each section in blue boxes.
- Examples of what to say to the patient are also included.
- ***Remember to complete the checklist at the end of each appointment.***
- All of the materials you will need to deliver the adherence solutions can be found in the Solutions Folder (including laminated copies of the solution guides).
- You will also need access to a computer with printing facilities (black and white ink).

Appointment information

Please complete for each appointment:

Date of Appointment 1:

Time started:

Time finished:

Carer/relative present (please tick) ☐

Date of Appointment 2:

Time started:

Time finished:

Carer/relative present (please tick) ☐

Date of Appointment 3:

Time started:

Time finished:

Carer/relative present (please tick) ☐

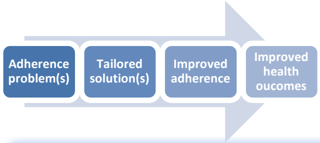

Section 1: Medication Details

Reminder instructions for pharmacists

- Please complete blue parts of this form BEFORE APPOINTMENT 1 using the PMR.
- If there are any discrepancies, the list should be confirmed with the GP surgery before the appointment.
- The grey parts of this form should be completed DURING APPOINTMENT 1. Part of this section is linked with Question 1 (Patient's knowledge) in the ID-MAP Tool on the next page (*Section 2*).
- Use Question 1 to confirm, with the patient, that the medication list is accurate and up-to-date.

**Complete BEFORE Appointment 1**		**Complete DURING Appointment 1**					
**Prescribed medication details** (i.e. **long-term** regular and when required medications)		**Details confirmed** *(Please tick)*		Linked with Question 1 **(Patient's KNOWLEDGE)** in ID-MAP Tool *Mark box with a question mark (?) if a knowledge issue is identified*			
**Medication** (Name, Form, Strength)	**Directions** (Dose, Frequency)	**By Patient**	**By GP** *(if there is a query)*	**What** *It is called*	**Why** *they take/ use it*	**When** *they take/ use it*	**How** *they take/ use it*

**Other medications** (e.g. hospital dispensed or OTC medications) **Complete DURING Appointment 1**	
**Medication** (Name, Form, Strength)	**Directions** (Dose, Frequency)

Date completed:		Signature:
List confirmed with GP if there is a query *(please tick)* ☐	Date:	Signature:
Date list confirmed with patient:		Signature:

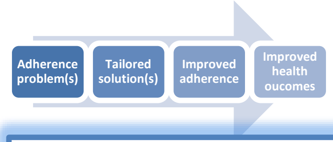

Section 2: ID-MAP Tool

Reminder Instructions for pharmacists

- The questions in this tool will help you identify individual adherence problems. Use prompts if necessary.
- **IMPORTANT:** Question 1 should be asked in conjunction with the medication list on the previous page. Ask the WHAT, WHY, WHEN and HOW aspects of this question for EACH medication. Place a question mark **(?)** in the corresponding box in Section 1 if a knowledge issue is identified.
- For all seven questions in this ID-MAP Tool, tick either the YES or NO box for each "Adherence problem" statement in the left hand column and make brief notes of the key issues in the space provided (e.g. which medication/s, specific reason for forgetting, misconceptions about their medicines etc.)
- Ignore the adherence solution(s) column during Appointment 1.

What to say to the patient: *"As I mentioned previously, the aim of this discussion is to find out a bit more about how you take or use all of your medicines and to see if there is anything we can do to help you get the most from them. If it is ok with you, I would like to start by asking you a few questions about the medicines you are currently taking (or using). If you don't mind, I will take some notes as we go along."*

Question 1 (Patient's Knowledge) **Use in conjunction with medication list (Section 1) **

What • *"Can you tell me WHAT medications you are currently taking (or using)?" (Confirm each medication in list)*

Why • *"Do you know WHY you are taking/using this medication?" (Go through each medication in list)*

When • *"WHEN do you take/use this medication?" (Go through each medication in list)*

How • *"Do you know HOW to take this medication/use this device?" (Ask as appropriate for each medication in the list)*

Adherence problem	YES	NO	Notes	Adherence solution(s)
Lacks knowledge of **WHAT** medications they are taking/using	☐	☐		1. Where knowledge gaps are identified, explain what each medication is and why they need to take/use it<br>2. Emphasise this by directing the patient to the back page of their medication diary **(Solution A)**
Lacks knowledge of **WHY** they are taking/using their medication(s)	☐	☐		
Lacks knowledge of **WHEN** to take/use medication(s) or incorrect timing	☐	☐		1. Give verbal feedback on what they are doing incorrectly<br>2. Explain importance of taking/using medications at correct time/following any special instructions/ correct dose<br>3. Emphasise this by directing the patient to the back page of their medication diary **(Solution A)**
Taking/using incorrect dose or other medication error(s)	☐	☐		
Lack of or incorrect knowledge on **HOW** to take their medication(s)/ use devices	☐	☐		1. Give verbal feedback on what they are doing incorrectly<br>2. Emphasise importance of correct use and if possible, write this in the special instructions section on the back page of their medication diary **(Solution A)**

Question 2- (Routine/organisational barriers): ***"Can you describe your typical daily medicine routine or tell me how you go about taking/ using your medicines when you are at home?"***

Prompt- *"Do you set your medications out in advance?"*

Prompt- *"Do you ever forget to order your medications and have to go without them?"*

Adherence problem	YES	NO	Notes	Adherence solution(s)
Lack of an effective **routine**	☐	☐		1. Advise to link medication use to routines already in place e.g. meals/ brushing teeth/TV show **(Solution B)**<br>2. Emphasise the importance of using the medication diary every day as they develop their routine **(Solution A)**
Lack of **planning** (e.g. forgets to order medication)	☐	☐		1. Emphasise the importance of using the medication diary every day as they develop their routine **(Solution A)**<br>2. Advise patent to mark re-order dates on the diary provided (e.g. a star beside medications that need re-ordered that week)

Question 3-(Practical barriers): ***"Do you have difficulty taking (or using) any of your medicines?"***

Prompt- *"Do you have difficulty opening the packaging/ using your devices/ swallowing the medication/ reading or understanding the labels?"*

Adherence problem	YES	NO	Notes	Adherence solution(s)
Difficulty opening medication **packaging**<br><br>Difficulty with **administration/ accessing medication**	☐<br><br>☐	☐<br><br>☐		1. Provide/source appropriate containers or administration aids if possible (e.g. inhaler aids) **OR** refer to GP/arrange appropriate solution (example if MDS is required)<br>2. Put a note on the PMR to ensure on-going support **OR** Complete GP communication form **(Solution B)**
Problem with the **formulation** (e.g. difficulty swallowing)<br><br>Problem with **regimen** (e.g. too complex)	☐<br><br>☐	☐<br><br>☐		1. Use your professional judgement to decide on the best course of action. Refer to GP if it is affecting adherence<br>2. Complete GP communication form **(Solution B)**
Difficulty **reading or understanding labels**	☐	☐		1. Provide patient with LARGE PRINT labels that are clear/easy to understand<br>2. Put a note on the PMR **(Solution B)**
Other<br>(Please document)<br>____________________	☐	☐		1. Decide on an appropriate course of action **OR** refer to GP **(Solution B)**<br>2. Complete GP communication form

Question 4- (Social support): *"Does anyone at home help you with your medicines?"*

Prompt- *"Do you have any friends or relatives that might be able to help?"* (For example, help you to order your medicines, collect prescriptions/medicines or remind you to take/use your medicines)

Prompt- *"Could the pharmacy staff do anything to help you?"* (For example, prescription collection or delivery)

Adherence problem	YES	NO	Notes	Adherence solution(s)
Current lack of social support from **family/friends**<br><br>Current lack of social support from **healthcare professionals**	☐<br><br>☐	☐<br><br>☐		Encourage patient to ask a relative/friend to remind them or help organise medications (order, collect, dispose)<br>**AND/ OR**<br>Arrange for social support from pharmacy staff/other healthcare professionals e.g. prescription collection or delivery **(Solution B)**<br>***Note-*** *the patient may not want additional social support*

Question 5 (Forgetting): *"Think back to the last time you forgot to take (or use) your medications, can you tell me why you think you forgot to take (or use) them?"*

Prompt- *"Were you out of the house, on holidays or just busy doing other things?"*

Prompt- "*Does someone usually remind you or do you have something else in place to help you remember, for example an alarm or mobile phone reminder?"*

Adherence problem	YES	NO	Notes	Adherence solution(s)
**General difficulties** remembering<br><br>Forget when **busy** with other household tasks<br><br>Lack of routine<br><br>Lack of reminder strategies	☐<br><br>☐<br><br>☐<br><br>☐	☐<br><br>☐<br><br>☐<br><br>☐		1. Emphasise the importance of the daily medication diary **(Solution A)**<br>2. Advise to link medication use to routines already in place e.g. meal times/ brushing teeth/TV programmes<br>**AND/OR** Advise storage of medications in a visually prominent place<br>**AND/OR** Encourage the use of reminder stickers in visually prominent places<br>**(Solution B)**
Forget when not at home/**out-of-routine** (e.g. day trips, meetings, appointments, holidays)	☐	☐		1. Emphasise the importance of the daily medication diary **(Solution A)**<br>2. Advise patient to mark travel dates on the diary (e.g. with the letter H)<br>**AND/OR** place a reminder sticker **(Solution B)** in their own travel itinerary when planning their holiday
Forget when **no-one** is there **to remind them** (e.g. spouse, family)	☐	☐		1. Emphasise the importance of the daily medication diary **(Solution A)**<br>2. Encourage the use of reminder stickers in visually prominent places at home<br>**AND/OR**<br>Advise storage of medications in a visually prominent place **(Solution B)**<br>3. If patient lives alone, encourage them to get other relatives /friends involved in helping them (if possible) e.g. reminder phone calls **(Solution B)**

<u>Question 6- (Intentional non-adherence)</u>: ***"People sometimes miss taking (or using) their medicines for reasons other than forgetting. Have you ever cut back or stopped taking (or using) any of your medicines without telling your doctor?"***

If YES- <u>In relation to the medicine(s) you don't take (or use) as often as you should, can you tell me why?"</u>

Prompt- *"Are you experiencing, or worried about, side effects or long-term effects of your medicine(s)?"*

Prompt- *"Do you find the medication is not working for you?"*

Adherence problem	YES	NO	Notes	Adherence solution(s)
Patient **<u>makes decision</u>** to stop, cut-back or alter dose of medication(s) **<u>without informing GP</u>**	☐	☐		1. Give feedback on what they are doing incorrectly<br>2. Emphasise/discuss why the medication should not be stopped/altered<br>3. Provide/discuss 'Voicing concerns about your medication' leaflet **(Solution C)**
Has experienced **<u>side effects</u>**	☐	☐		1. Can the side effect(s) be managed? If not, refer to GP. Complete GP communication form **(Solution B)**<br>**<u>OR</u>** if side effects can be managed, reassure the patient and discuss any misconceptions **(Solution C)**<br>2. Provide/discuss 'Voicing concerns about your medication' leaflet **(Solution C)**
Worried about side effects and/or long-term consequences	☐	☐		
Thinks the medication **<u>isn't working</u>**	☐	☐		1. Inform them of the benefits/necessity of <u>ALL</u> their medications and discuss any misconceptions<br>2. Provide/discuss 'Voicing concerns about your medication' leaflet **(Solution C)**
Thinks one (or some) medications are **<u>less important</u>** than others	☐	☐		
**<u>Unsure of</u>** the **<u>consequences</u>** of non-adherence	☐	☐		1. Give feedback on what they are doing incorrectly<br>2. Explain consequences of non-adherence **<u>AND/OR</u>** discuss importance of not missing doses if it can be avoided<br>3. Provide/discuss 'Voicing concerns about your medication' leaflet **(Solution C)**
Thinks it is OK to miss a few days/doses of medication	☐	☐		
Believe **<u>generic medicines</u>** are less effective compared with branded medicines	☐	☐		1. Provide/discuss 'Voicing concerns about your medication' leaflet<br>2. Provide/discuss 'Generic Medicines Fact Sheet' **(Solution C)**
Concerns about generic medicines	☐	☐		

Question 7- (Patient's motivation): *"On a scale of 0 to 10, how important is it for you to take (or use) all of your medicines as prescribed?" (Show patient the scale below and circle score)*

If patient indicates a score of 7 or less on scale then ask: "What would motivate you to take (or use) all of your medicines as the GP has prescribed?"

Prompt- *"Is there anything that you would like to achieve from taking/using your medicines?"* (E.g. stay healthy/active, stay out of hospital, stay at home, be free from symptoms, reduced symptoms)

Adherence problem	YES	NO	Notes	Adherence solution(s)
Patient's **motivation** to take/use medications **could be increased** by setting a positive general health goal (*Tick NO if patient appears to be already highly motivated i.e. score of 8 or above on the scale below*)	☐	☐		Together with the patient, set an overall general health goal. ***(Note: this goal will be in addition to the patient's specific medication use goal/s)***. This goal should be focused on a positive outcome of taking/using their medicines e.g. stay out of hospital **(Solution D)**

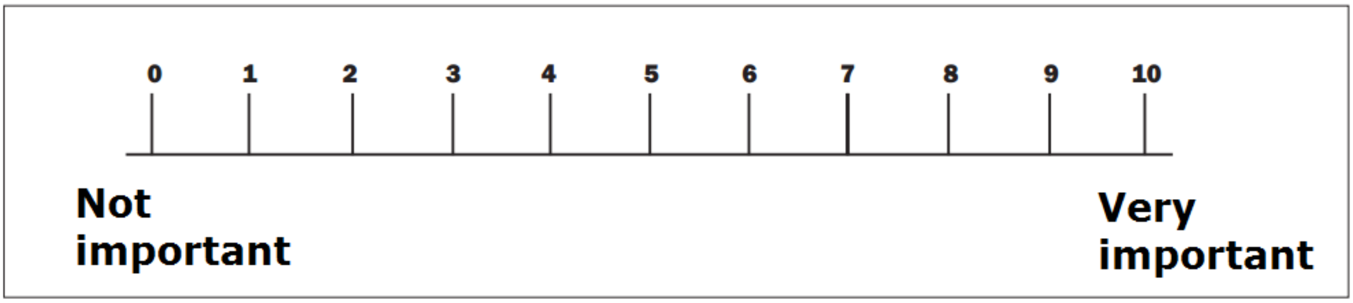

********This is the end of Appointment 1*********

What to say to the patient: *"Those are all the questions I have for you today. Is there anything you would like to ask me? The next stage is to look at what options we have to help you get the most from your medicines. I would like to arrange another appointment to take place in 1-2 weeks if that suits you? This will give me some time to prepare a medication diary for you and arrange other solutions that I think you might find useful. Would any particular day or time suit best?"*

Appointment 1 check list

Fill in this checklist at the end of Appointment 1

ALL blue boxes **MUST** be ticked. White boxes should be ticked if applicable.

**Section 1**- Medication Details section has been completed and confirmed as accurate	☐
**Section 2**- ID-MAP Tool has been completed (at least one problem identified)	☐
Urgent adherence problems have been resolved and GP contacted (if necessary)	☐
**Appointment 2-** Date and time arranged ____________________________	☐

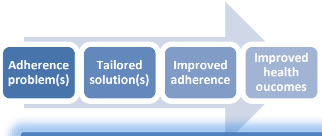

Section 3: Solution Summary (page 1 of 3)

Reminder instructions for pharmacists

- Go back through Section 2 and map adherence problems (red YES column ticks) across to adherence solutions.
- Note: ***All patients will receive Solution A*** *(Medication diary)* ***AND Solution D*** *(Goals and action plan sheet).*
- Please **tick any additional optional solutions** (B and/or C) required (on coloured boxes on page 10 & 11)
- A space for planning notes is provided for each adherence solution: **use a RED pen for planning notes.**
- Additional space is provided for any notes made during Appointment 2: **use a BLACK pen for additional notes**
- Prepare adherence solutions in advance (instructions and materials can be found in the Solutions Folder).
- Make brief summary notes on the adherence problems discussed at Appointment 1 (black box below). This will allow you to quickly recap on these discussions at the start of Appointment 2.

What to say to the patient: *"Thank you for coming along today. The purpose of today's appointment is to discuss things that might help you with taking (or using) your medicines. To start us off, I would like to recap on some of the things we discussed at the last appointment."*

Recap of discussions from Appointment 1 (Summary notes)

Space for additional notes

Solution Summary (page 2 of 3)

☑ **Solution A – Patient medication diary (compulsory)**

- *Prepare medication diary in advance using the confirmed list of medications (prescribed medications only).*
- *Provide **ALL PATIENTS** with medication diary during Appointment 2 and explain how to use it.*
- *Ask the patient to bring the diary along with them to their next appointment (at least four weeks later).*

☐ Please tick if additional verbal advice/feedback is required (e.g. knowledge gaps identified).

Planning notes:

Additional notes:

☐ **Solution B – Practical, reminder and social support options (optional)** *(tick if delivered)*

- *Tick possible options identified using the ID-MAP Tool and if necessary prepare these before Appointment 2.*
- *During Appointment 2, agree which option is most suitable (**Note**: may require more than one option).*
- *Provide resource(s)/make notes in PMR where necessary.*

Possible options	***Option agreed with patient***
[ ] Provide more suitable packaging ____	[ ]
[ ] Provide large print labels /clearer wording________________________________	[ ]
[ ] Link medication taking to other routines __________________________________	[ ]
[ ] Advise storage in a visually prominent place ______________________________	[ ]
[ ] Provide reminder stickers ____	[ ]
[ ] Social support plan (relatives/friends/healthcare team) __________________	[ ]
[ ] Other (please detail) ____	[ ]
[ ] Referral to GP (Fill in GP communication form: reason(s) ________________	[ ]

Planning notes:

Additional notes:

Solution Summary (page 3 of 3)

☐ **Solution C –Discussion around medication concerns (optional)** *(Tick box if delivered)*

- *Make planning notes in relation to issues that need to be discussed during Appointment 2.*
- *During Appointment 2, discuss these issues using the 'Voicing concerns about your medicines leaflet' as a visual aid.* ***Emphasise the health consequences of non-adherence.***
- *Complete section on the back of the leaflet ('Who to contact if you have concerns').*
- *If the patient has concerns about generic medicines, also provide/discuss 'Generic medicines fact sheet'.*

  ☐ *Please tick if the 'Generic medicines fact sheet' is also required.*

Planning notes (issues to discuss):

Additional notes:

☑ **Solution D – Goals and action plan sheet (compulsory)**

- *List possible medication use goals, general health goals (optional) and action plans in the planning section below in advance of Appointment 2.*

  ☐ *Please tick if an overall general health goal is also required (to help increase motivation)*
- *Purpose of setting goals and having an action plan should be explained during Appointment 2.*
- *Fill in the goals and action plan sheet for* ***ALL PATIENTS.*** *You may need to prompt them to set goals.*
- *Give top copy to patient. Keep duplicate copy for your records.*

Planning notes (potential medication use goal/s):

Examples*-take my blood pressure medicines every day/take my inhalers when I am out for the day*

Planning notes (potential general health goal if required):

Examples*- to stay well and in my own home*/to be free from night-time asthma symptoms

Planning notes (potential action plan/s):

Can include: details of new routine, exactly when/where to take, who to contact if concerned, who will provide support etc.

Examples- IF I am concerned, THEN I will speak to my pharmacist/ IF I see my medicines sitting on the kitchen bench, THEN I will take them

Additional notes:

*******This is the end of Appointment 2*********

<u>What to say to the patient:</u> *"Hopefully you have found today's appointment useful. Do you have any further questions you would like to ask?*

Please let me know if you have difficulties with the medication diary or anything else we discussed today. If any of your medicines change during the next four weeks, please contact me as soon as you can. We can make any changes needed to the diary if this happens.

I would like to arrange another appointment for four weeks' time if that suits you. We can find out how you got on with everything at this next appointment. Would any particular day or time suit best?

Appointment 2 check list

Fill in this checklist at the end of Appointment 2

ALL blue boxes **MUST** be ticked. White boxes should be ticked if applicable.

Section 3- Solution Summary pages have been fully completed ☐

Solution A- Purpose of the medication diary explained ☐

- **Personalised** medication diary provided to patient ☐
- Additional information provided **if** knowledge gaps were identified ☐
- Patient asked to bring completed medication diary to the next appointment ☐

Additional solution B provided if necessary ☐

- Recommended action taken/resources provided/advice given ☐

Additional solution C provided if necessary ☐

- Leaflet(s) discussed and completed if necessary ☐
- Patient given leaflet(s) to take home ☐

Solution D- Purpose of setting goals and having an action plan explained to the patient ☐

- Goals and action plan sheet completed ☐
- Top copy given to patient, bottom copy retained for records ☐

Appointment 3- Date and time arranged ___________________________ ☐

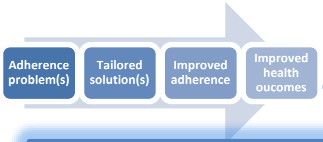

Section 4: Review of solutions/feedback

Reminder instructions for pharmacists

- At Appointment 3 you will find out how the patient got on with the recommended adherence solutions.
- You will need to review compulsory solutions A and D.
- You will also need to review optional Solutions B and/or C if these were delivered.
- Indicate *"N/A"* or *"Not applicable"* in the notes section if a solution was **not** delivered.
- The table below will help guide this appointment. Make brief notes about your discussions.

What to say to the patient: *"Thank you for coming along again today. The purpose of today's appointment is to review how you got on with everything we discussed at the last appointment. If it is okay with you, I would like to start by taking a look at your medication diary—did you bring this along with you?"*

Solution	Steps to review it	Notes
**Solution A: Medication diary**	1. **Did they use the daily medication diary?** If not, discuss why. Discuss reasons for missed doses.<br>2. **If possible, give the patient feedback on their adherence behaviour based on the diary**. Was there any improvement or is there still some room for improvement?<br>3. Ask if they are happy for you to **keep the diary for the research team to examine!** You can print out a separate list of medications if the patient would like this for their own reference. ***Ensure this is kept up-to-date***	
**Additional solution B** (practical, reminder, social support)	1. **Did the recommendations help?** *(Refer to page 10 & 11 for what was recommended)*<br>2. **Are any further actions required?** If so, please make a note of these.	
**Additional solution C** (discussion around medication concerns)	1. **Did they find the leaflet(s) helpful?** Do they have any more concerns that they wish to discuss?<br>2. **Are they aware of the health benefits** of taking/using their medications as prescribed and the possible consequences of non-adherence?	
**Solution D: Goals and action plan**	1. **If an overall general health goal was set, is this still their motivation** for taking/using their medicines?<br>2. **Did the patient meet their medication use goal(s)?** If the medication diary indicates that they did not fully meet their goal(s), then discuss the possible reasons why.<br>3. **Complete the 'Review of goal' section on Goals and action plan sheet** for each medicine use goal by ticking the relevant option (goal met or goal not met).	

*****This is the end of the ID-Map intervention******

__What to say to the patient:__ *"Thank-you for coming along to all three appointments; I hope you have found them useful. This is the last official appointment but if you have any more questions about your medicines please feel free to call into (or telephone) the pharmacy at any time. I would be happy to answer any questions you may have. To find out more about what you thought of the appointments, the research team will telephone you over the next few days; does any particular time suit you best?"*

Appointment 3 checklist

Fill in this checklist at the end of Appointment 3

ALL blue boxes **MUST** be ticked. White boxes should be ticked if applicable.

**Section 4-** Solution review table has been completed/notes made	☐ (blue)
-Medication diary has been returned by the patient	☐ (blue)
-Medication list supplied (if requested by patient)	☐
-Further recommendations made (if necessary)	☐
Patient advised that the research team will follow-up with them in the next few days	☐ (blue)
Most appropriate time for research team to telephone ___________________________	☐ (blue)

Queen's University
Belfast
